# Nanophosphors-Based White Light Sources

**DOI:** 10.3390/nano9071048

**Published:** 2019-07-22

**Authors:** Maura Cesaria, Baldassare Di Bartolo

**Affiliations:** 1 Department of Mathematics and Physics “Ennio De Giorgi”, University of Salento, prov.le Via Arnesano, 73100 Lecce, Italy; 2 Department of Physics, Boston College, Chestnut Hill, MA 02215, USA

**Keywords:** solid state lighting, white-light emission, size-dependent spectroscopy, nanophosphors, un-doped white-light emitters

## Abstract

Miniaturization requests and progress in nanofabrication are prompting worldwide interest in nanophosphors as white-emission mercury-free lighting sources. By comparison with their bulk counterparts, nanophosphors exhibit reduced concentration quenching effects and a great potential to enhance luminescence efficiency and tunability. In this paper, the physics of the nanophoshors is overviewed with a focus on the impact of spatial confinement and surface-to-volume ratio on the luminescence issue, as well as rare earth-activated multicolor emission for white light (WL) output. In this respect, the prominently practiced strategies to achieve WL emission are single nanophosphors directly yielding WL by means of co-doping and superposition of the individual red, green, and blue emissions from different nanophosphors. Recently, a new class of efficient broadband WL emitting nanophosphors has been proposed, i.e., nominally un-doped rare earth free oxide (yttrium oxide, Y_2_O_3_) nanopowders and Cr transition metal-doped garnet nanocrystals. In regard to this unconventional WL emission, the main points are: it is strictly a nanoscale phenomenon, the presence of an emitting center may favor WL emission without being necessary for observing it, and, its inherent origin is still unknown. A comparison between such an unconventional WL emission and the existing literature is presented to point out its novelty and superior lighting performances.

## 1. Introduction

Solid-state lighting based on light emitting diodes (LEDs) is a rapidly growing market that is progressively replacing the old lighting lamp technologies (incandescent light bulbs, compact fluorescence lamps, and high-intensity discharge lamps). The underlying reason is that, while the efficiency performances of conventional white light (WL) sources (lamps) seem to have reached an upper limit [1], solid-state WL-emitting LEDs are a generation of lighting sources with mature technology and are particularly interesting for achieving mercury-freedom, better power output, and small volumes in integrated electronics [2,3]. In particular, in the field of indoor and outdoor lighting, most efforts are being focused on artificial sources with high efficiency, low power consumption, durability, thermal and chromatic stability, fast switching, small size, high color rendering, price competitiveness, environmental friendliness, and, very importantly, sunlight-like WL emission (which is the most comfortable to the human eye).

The breakthrough in the emergence and development of LED-based WL sources dates to the development of red LEDs and the invention of bright-blue LEDs by Nakamura et al. in the mid-1990s [4], when efficient WL production was demonstrated by mixed blue-yellow emission resulting from a blue emitting (λ ≈ 440−460 nm) InGaN LED chip exciting a yellow-emitting down-converting Ce-doped yttrium aluminum garnet (Y_3_Al_5_O_12_) crystal [5]. Ce^3+^-activated Y_3_Al_5_O_12_ features broadband yellow emission, efficient absorption for blue light (420−480 nm), quantum efficiencies larger than 90%, high thermal quenching temperatures (~700 K), and fast decay rates (~63 ns) [6,7]. The intentional addition of Mg^2+^ and Si^4+^ ions enables a shift of the emission maximum of Ce^3+^-activated Y_3_Al_5_O_12_ to around 600 nm, which leads to a warm WL emission under pumping with a blue emitting LED [8].

Actually, most high-brightness white LED sources use a (blue near-ultraviolet or ultraviolet (UV)) diode that pumps a single or a combination of luminescent materials termed phosphors. Such phosphor-converted LEDs have gained tremendous achievements into various applications and promise to further expand their application fields. As a general remark, fundamental and applicative research efforts to improve performances of WL LED sources are currently mainly devoted to nanophosphors (i.e., nano-sized phosphors) [9,10,11,12] due to changes of several properties related to scaled-down size and opportunities of engineered functionalities for the desired application. For instance, an increasing trend to miniaturization favored by the progress in nanotechnology and the development of nano-medicine have prompted interest in nanophosphors as luminescent markers for imaging in medical diagnosis and therapy as well as multiplexed biological labeling. Such applications take advantage from up-conversion luminescence, large anti-Stokes shifts (up to 500 nm), excellent photo-stability, high luminescence quantum efficiency, long luminescence lifetime, narrow emission lines, high color purity, removal of UV excitation-induced photo-damage to biological samples, quantum cutting, and absence of photo-bleaching and photo-blinking in nanophosphors [9,10,13,14,15].

It is worth stressing that the peculiar spectroscopy of the nanophosphors does not result from quantum size effects which dominate the emission spectra of semiconductor quantum dots. Instead, the active role is played by size-scale, doping, site symmetry, phase of the crystalline matrix, dopant-ligand distance and strength of the coordination, as well as influence of the surfaces [16].

In this review paper, we overview the spectroscopy of the nanophosphors with particular attention to the effects of spatial confinement and surface-to-volume ratio on their emission performances in terms of electron-phonon dynamics, luminescence quenching, confinement of dopants, and tuning of multicolor emission for the generation of WL emission and dynamical characteristics (emission decay and rise time, line broadening and line shift). Moreover, we present and discuss the observed occurrence of unconventional WL emission, where “unconventional” refers to a new class of efficient broadband white-light-emitting nanophosphors consisting of nominally un-doped rare earth (RE)-free oxide nanopowders [17] and transition-metal (TM)-doped nanoscale hosts [18] excited by monochromatic infrared-excitation. By comparison with the literature, the actually available strategies to obtain WL emission mainly consider RE dopants and single-phase compounds containing REs as stoichiometric components.

Since a huge amount of literature is already available dealing with the general properties of phosphors and their spectroscopy, the main intent of this review paper is not to provide a didactic overview of the field again. Instead, we overview the physics of nanophosphors, which is a fascinating and still not completely understood research field, and introduce the reader to a very recent unconventional phenomenon in the field of WL emission. In this spirit, the presented discussion and examples aim at summarizing the existing knowledge in such a way to point out the main differences, achievements, and challenges in the field of nanophosphors as compared to bulk phosphors. While keeping in mind this purpose and in order to make the readership of this review contribution open to non-specialist readers, we clearly indicate nomenclature and provide self-consistent knowledge of basic concepts, definitions, and spectroscopic mechanisms mentioned throughout the paper. For more informative fundamental knowledge, we suggest a preliminary reading of Reference [16] to the reader new to the field of the spectroscopy of phosphors.

The paper is organized as follows: after a general introduction about the emission mechanisms and physics of phosphors and RE dopants, a section will be devoted to the spectroscopy of nanophosphors and, in addition, a section dealing with the experimental findings related to the observed unconventional WL emission will be presented. In this respect, main points resulting from the experimental evidence are: first, such unconventional WL emission is a nanoscale phenomenon, second, its occurrence is strictly related to the host matrix because, although doping can favor WL emission, doping is not needed, and third, its inherent origin is still unknown, despite an attempt of interpretation of the experimental findings [19]. By comparison with the existing scenario, challenging questions to be addressed at the fundamental level will be pointed out and discussed.

## 2. Phosphors and Phosphor-Converted Solid-State Lighting Sources: Background

### 2.1. Phosphors and Luminescence Mechanisms

Inorganic phosphors are solid-state efficient luminescence materials that can be involved in conventional down-conversion processes (i.e., emission of photons with lower energy than the exciting photons) as well as up-conversion processes (i.e., emission of photons with energy exceeding the exciting photon energy by 10–100 k_B_T) [20,21].

**Figure 1:**
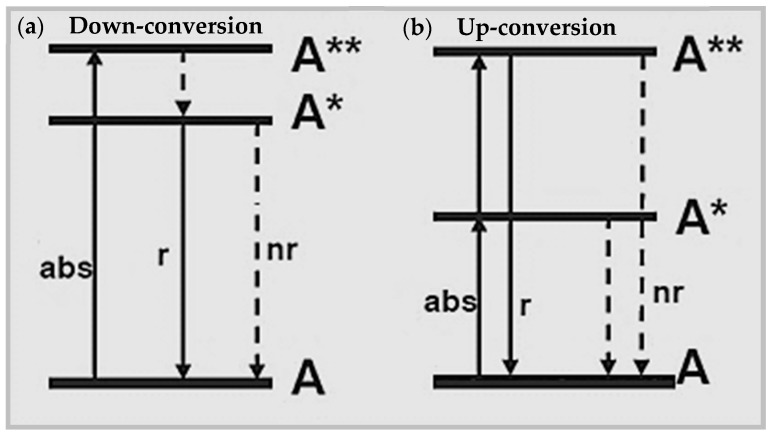
Schematics of (**a**) down-conversion emission and (**b**) up-conversion emission resulting from energy absorption (abs) by an element A with excited states A* and A**. Labels “r” and “nr” refer to radiative and non-radiative decay-channels, respectively.

Figure 1 shows a schematics of a down-conversion process (Figure 1a) and an up-conversion process (Figure 1b) following energy absorption (“abs” label) between the ground state and the excited states (referred to as A* and A**) of an element A. Decay channels of the absorbed energy can be radiative (“r” label) through down-conversion and up-conversion emission or non-radiative (“nr” label), typically heat transfer, which compete with luminescence and deteriorate the luminescence yield and efficiency (number of photons emitted divided by the number of photons absorbed).

Phosphors can have different composition according to the following main classification:

- thermally and chemically stable crystalline materials that inherently contain luminescent centers,
- optically inactive materials (termed host crystals) doped with optimized concentrations and kind of luminescent ions (activators, sensitizers, activator–sensitizer pairs), and
- defect-related luminescent materials that emit under proper concentration of the defect and/or reaction conditions.

The host material of a phosphor is usually a wide band-gap material (e.g., oxides, nitrides, and sulfides) that fulfills a stringent requirement such as low lattice phonon frequencies [22] to improve the emission efficiency and reduce non-radiative losses [23]. On the other hand, the host crystal plays a role in determining dopant-ligand coordination and spatial distance as well as site-symmetry, which is an important issue depending on the dopant nature and bulk versus nanoscale regime. As it will be detailed, the host material may affect luminescence properties such as emission color, quantum conversion efficiency, and thermal quenching of optical transitions involving d electronic orbitals. Notably, as the lanthanide-ligand bonds are more ionic than the transition metal-ligand bonds, more coordination situations are possible in the case of RE elements and this corresponds to more symmetries.

**Figure 2:**
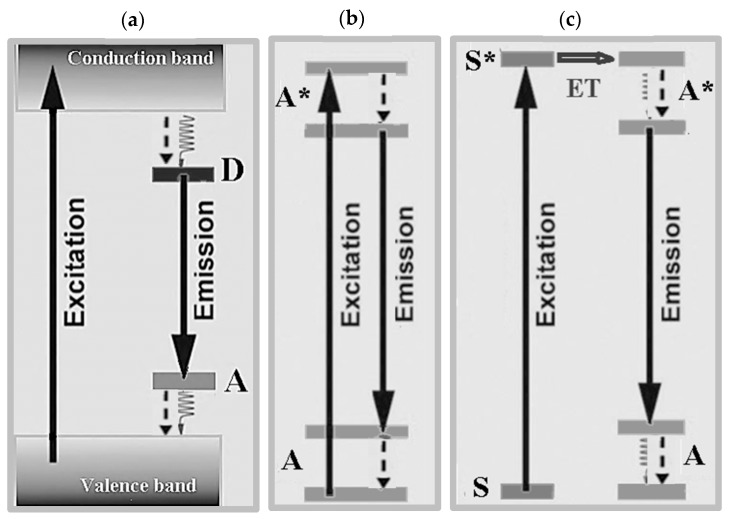
(**a**) Sensitization of the host lattice. (**b**) An activator element (A) gets excited to a state A* following energy absorption and emits radiatively through down-conversion. (**c**) The energy absorbed by a sensitizer S having an excited state S* is transferred to an excited state of a nearby activator dopant (A*) that emits radiatively. Dashed lines refer to non-radiative energy relaxation.

Radiative emission of phosphors can result from two main classes of processes, which are, sensitization by the host lattice or transitions due to dopants termed “activator” (A) if acting as emitting centers and “sensitizer” (S) if their energy levels let transfer energy to the emitting activator ions [24]. In the former situation (Figure 2a), band-to-band excitation by effective photon absorption generates electron–hole pairs (typically excitons under UV excitation) and then conduction excited electrons relax to donor levels (“D” label) and holes transfer to acceptor states (“A” label) rather than recombining radiatively. Hence, fluorescence by sensitization by the host lattice stems from levels of the donor–acceptor pair. In the case of luminescence between discrete energy levels of emitting centers without the participation of the host material, the simplest situation occurs when an activator dopant element (A) gets excited to a state A* following energy absorption and emits radiatively through down-conversion processes (Figure 2b). Fluorescence with tunable color output of an activator element can be induced indirectly by energy transfer from a directly excited sensitizer to the excited state of a nearby radiatively emitting activator (Figure 2c).

An efficient sensitizer is required to have strong and broad absorption/emission favoring energy transfer towards the distribution of the absorption lines of the acceptor ion. Among available sensitizers and activators, the Er^3+^-Yb^3+^ pair is particularly suitable under excitation at 980 nm [25]. Fluorides usually exhibit low phonon energy of (350 cm^−1^) and, for instance, hexagonal NaYF_4_ crystal co-doped with Yb^3+^-Er^3+^ pairs is an efficient up-conversion phosphor under excitation at 980 nm [26].

The above considerations shed light on the important role played by the energy transfer processes, which can be classified into resonant, semi–resonant, and double resonant energy transfer. In the former case, energy transfer occurs from donor to acceptor with slightly lower-lying energy levels in such a way to make unlikely backward energy transfer from acceptor to donor. Semi–resonant and double resonant energy transfer are examples of non-resonant energy transfer. For semi–resonant processes, also termed phonon-assisted energy transfer, several phonons bridge the small energy mismatch between the donor and acceptor ions, and luminescence quenching may result due to the phonon-related radiative losses.

For double resonant energy transfer, two luminescent centers have energy levels at the energy E, the third center has an excited state at energy 2E, and the process, that works under the assumption that the three centers are closely spaced, can be described by the formula D* + A + A ↔ D + A* + A*, according to the nomenclature of the excited states (i.e., addition of an asterisk) [24]. The efficiency of an energy transfer process depends on the spectral overlap of the sensitizer–acceptor pair, the lifetime of the sensitizer in the absence and in the presence of the acceptor ion, and the relationship between the sensitizer–acceptor distance and a characteristic distance where the probability of energy transfer from a sensitizer center to an activator center equals the probability of radiative emission from the sensitizer [27].

An energy transfer process in which the decrease of energy of a center causes the excitation of a neighboring center is termed cross-relaxation, and may take place between identical centers or two different centers with matched pairs of energy levels.

**Figure 3:**
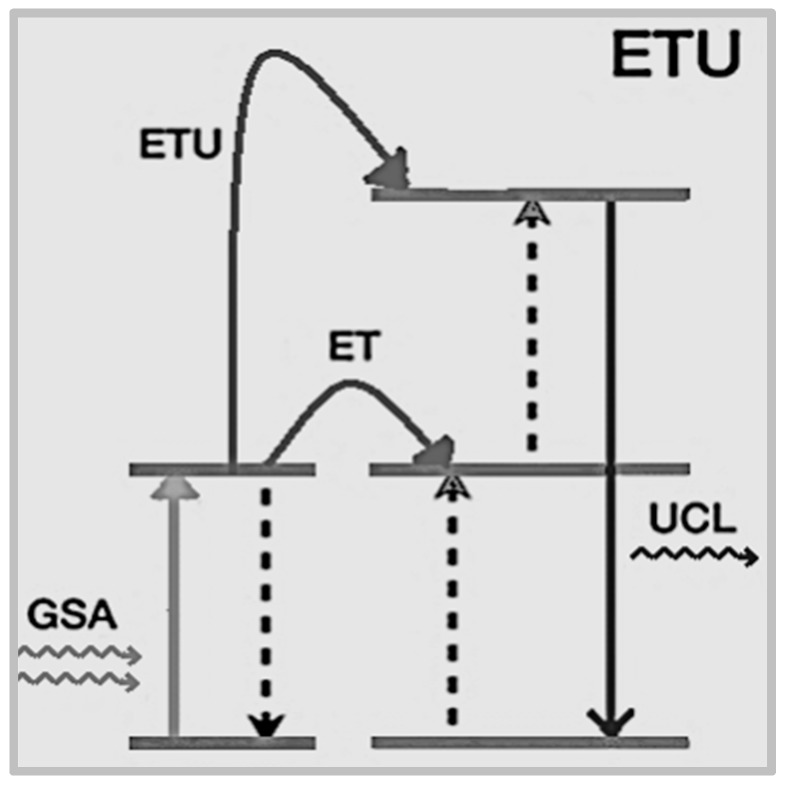
Schematic diagram of an energy transfer up-conversion (ETU) process involving ground state absorption (GSA), energy transfer (ET) between emitting centers, and up-conversion luminescence (UCL).

Energy transfer by up-conversion is a process resulting from the combined effect of energy transfer and up-conversion (Figure 3) when a sequence of energy transfer processes involves centers at different sites and a center is in an excited state: under photon excitation one center is excited by ground state absorption (GSA) and its excited state can transfer energy to a higher-lying energy level of second center yielding up-conversion luminescence.

### 2.2. Dopant Emitting Centers

The above general overview points out that luminescence at a designed color output requires that both the absorbed energy could be channeled to discrete states of emitting centers and effective interplay of luminescence processes occurs. The dopant commonly used in phosphor materials are RE and TM elements, which can be classified according to their absorption electronic transitions, as dictated by their electronic configuration.

**Figure 4:**
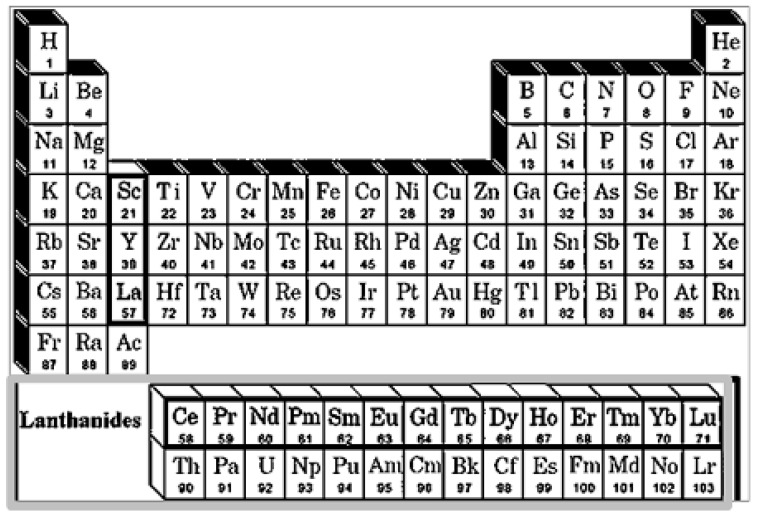
Periodic table of elements.

Actually, most phosphors incorporate RE elements that include, as the periodic table of elements in Figure 4 shows, scandium (Sc), yttrium (Y), lanthanum (La) and the 14 lanthanide elements (cerium (Ce), praseodymium (Pr), neodymium (Nd), promethium (Pm), samarium (Sm), europium (Eu), gadolinium (Gd), terbium (Tb), dysprosium (Dy), holmium (Ho), erbium (Er), thulium (Tm), ytterbium (Yb), and lutetium (Lu) with atomic number Z ranging from 58 to 71 [28].

A peculiarity of RE ions is their characteristic sharp emission energies related to their electronic configuration [Xe]4f^N^, where N refers to the number of electrons in the f-shell. Trivalent lanthanide ions have an outer electronic configuration 5s^2^5p^6^4f^N^ where N ranges from 1 (Ce ion) to 13 (Yb ion) and the valence electrons 4f^N^ are shielded from interactions with the chemical environment (host crystal lattice and ligands) by means of the 5s^2^5p^6^ outer less-bounded configuration.

According to the structure of their energy levels, RE elements can be involved in intra-configurational (i.e., 4f–4f) transitions with sharp line-like emission and inter-configurational (i.e., 4f–5d) transitions with broadband absorption and emission spectra [29,30]. By comparison with the parity forbidden intra-configurational transitions, the 4f^N^-4f^N−1^5d^1^ optical transitions have high radiative emission probability and short lifetime (tens of nanoseconds) [31,32].

The characteristic emission lines of all lanthanides in the range 0–40,000 cm^−1^ are assigned by the so-called “Dieke diagram” [33] that reports on the energies of the states of the trivalent RE ions indicated by the energy level of the corresponding free-ion (^2S+1^L_J_, spin (S), orbital (L) and angular (J) momentum quantum numbers) given by the center of gravity of each J-multiplet. Among emitting levels of lanthanides, there are ^4^G_5/2_ for Sm^3+^, ^5^D_0,1,2,3,4_ for Eu^3+^, ^5^D_3,4_ for Tb^3+^, ^5^S_2_ for Dy^3+^, ^5^S_2_ and ^5^S_5_ for Ho^3+^,^2^H_9/2_,^4^S_3/2_, ^4^F_9/2_ for Er^3+^, and ^1^D_2_, ^1^G_4_ for Tm^3+^. Among commonly exploited radiative transitions of trivalent lanthanides, there are: ^4^G_5/2_ → ^6^H_5/2,7/2,9/2_ of Sm^3+^, ^5^D_0,1,2_ → ^7^F_0,1,2,3,4_ of Eu^3+^, ^5^D_3,4_ → ^7^F_2,3,4,5,6_ of Tb^3+^, ^4^F_9/2_ → ^6^H_15/2,13/2_ of Dy^3+^, ^5^F^4^, ^5^S^2^ → ^5^I^8^ and ^5^I_4_ → ^5^I_8_ of Ho^3+^, ^2^H_11/2_, ^4^S_3/2_, ^4^F_9/2_ → ^4^I1_5/2_ of Er^3+^, as well as ^1^D_2_ → ^3^F_4_ and ^1^G_4_ → ^3^F_4_,^3^H_6_ of Tm^3+^. For instance, Eu^3+^ can yield red color emission with orange-red/red emission arising from the characteristic ^5^D_0_ → ^7^F_1_ (555 nm) and ^5^D_0_ → ^7^F_2_ (614 nm) electronic transitions. Dy^3+^ can simultaneously emit blue (483 nm, ^4^F_9/2_ → ^6^H_15/2_ transition) and yellow (584 nm, ^4^F_9/2_ → ^6^H_13/2_ transition) colors. Yb^3+^ ions have a simple distribution of electronic levels and, due to the matching with the exciting wavelength 980 nm of laser diodes, the most commonly exploited transition is ^2^F_7/2_ → ^2^F_5/2_. The excited states of Yb^3+^ ions are also suitable for energy transfer processes to the energy matching excited levels of Er^3+^, Tm^3+^, and Ho^3+^. The RE elements Tm^3+^, Er^3+^, or Yb^3+^ yield sharp-line red/green/blue emission, following absorption of Yb^3+^ and energy transfer to Er^3+^/Yb^3+^ levels. Tm^3+^ shows strong host-dependent blue emission (488 nm, ^1^D_2_ → ^3^F_4_ transition) and characteristic weak red emission (697 nm, ^1^G_4_ → ^3^F_4_ magnetic dipole transition).

A comprehensive classification and theoretical basis of the 4f–5d absorption and emission energies of divalent and trivalent lanthanides elements used as dopants of more than 1,000 inorganic compounds was provided by Dorenbos [34,35,36].

**Figure 5:**
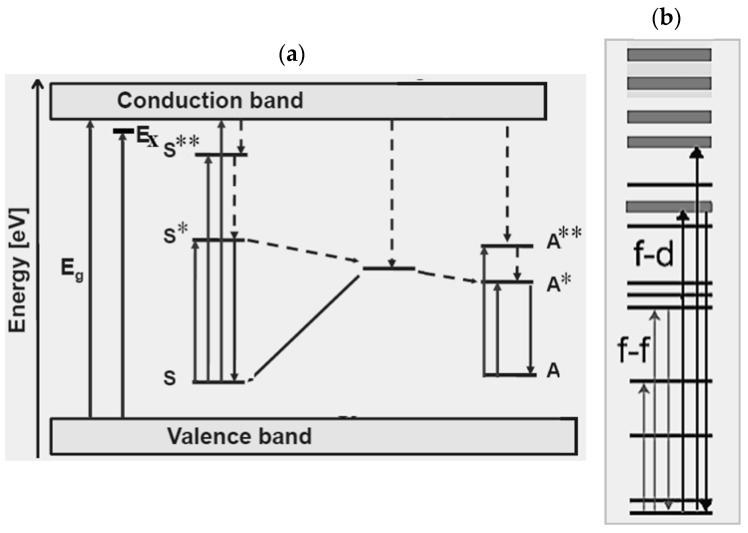
Characteristic luminescent processes occurring in a rare earth (RE)-doped host crystal. (**a**) Shows band-to-band absorption through the gap with energy gap Eg, generation of an exciton with energy Ex, absorption, luminescence and energy transfer processes related to sensitizer (S)-activator (A) pairs. The continuous and dashed lines refer to radiative and non-radiative transitions, respectively. The asterisk indicates excited states. (**b**) Sketches processes where an electron is excited from the lanthanide’s occupied 4f orbitals to its unoccupied 4f or 5d orbitals. The shaded upper region is the conduction band.

To summarize, Figure 5 depicts the characteristic luminescent processes occurring in inorganic phosphors composed by a host crystal including RE-emitting centers. Figure 5a shows band-to-band absorption through the gap with energy gap Eg, generation of a bounded electron–hole pair (an exciton with energy Ex), absorption, luminescence, and energy transfer processes related to sensitizer (S)-activator (A) pairs. The continuous and dashed lines refer to radiative and non-radiative transitions, respectively. The asterisk indicates excited states. Figure 5b sketches processes where an electron is excited from the lanthanide’s occupied 4f orbitals to its unoccupied 4f or 5d orbitals. The shaded upper region is the conduction band.

Rare earth ion-activated phosphors yielding up-conversion emission [20] by means of two or more photon absorption and energy transfer processes are widely used in solid-state lighting LED sources [37]. Since most activators, such as Er^3+^ and Tm^3+^, have complex energy level sequences, the assistance of phonons is necessary to observe up-conversion whenever the excitation light cannot exactly match with the up-conversion transitions. In up-conversion phosphors, energy losses can be due to both non-radiative relaxations (thermal losses) and competition between up-conversion luminescence and down-conversion transitions. In general, the main factors affecting the up-conversion strength are the photon energy and the symmetry of the host materials, the doping amount of activators and sensitizers, and the lattice constants.

### 2.3. Phosphor-Converted White-Light Emitting Diodes (LEDs)

**Figure 6:**
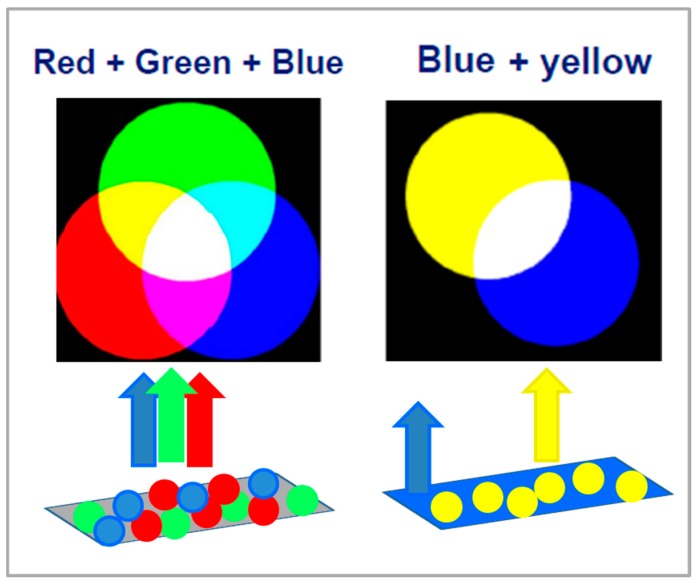
Most common principles used in white light emitting LEDs: (left panel) a (near-)ultraviolet (UV) emitting LED combined with RGB emitting phosphors and (right panel) a blue-emitting LED irradiating a yellow phosphor.

Since a LED is inherently a single-color emitter, WL emission from LED-based devices requires strategies that overlap/mix in a balanced way either two complementary colors or three primary colors (red (R), green (G), and blue (B)). For this purpose, the richness of color outputs and transition processes of RE elements make them key components of phosphors. Technically, LED WL-sources can consist of multiple LED chips (the so-called multiple LED approach), a (near-)UV emitting LED combined with RGB phosphors (Figure 6, left panel), and a blue-emitting LED plus a yellow phosphor (Figure 6, right panel) that can be replaced by a mixture of green and red phosphors with similar emission profiles [3,38,39,40]. Despite the advantages of using only LEDs (without phosphors) due to the relatively narrow emission bands, practical disadvantages of the multiple LED approach are more complex electronics, current- and temperature-dependent color shifts, high production costs, and the request of combining at least four LEDs. For these reasons as well as more flexible design opportunities, currently, most of the commercially available LED-based WL sources rely on phosphor-converted LEDs, i.e., on combining a single LED light source with one or more phosphors.

In phosphor-converted LED devices using a blue emitting LED, the phosphor converter is directly packed on the blue LED in such a way that the absorption of blue light by the phosphor yields yellow emission that, combined with the transmitted blue light, leads to WL luminescence (Figure 6, right panel).

Because of a missing red component, the chromatic performances of the emitted WL are poor. To overcome this drawback, yellow emitting phosphors are mixed with green to red emitting phosphors under blue light excitation [41]. Notably, as the naked eye is not sensitive to red emission beyond 650 nm, broadband emission red phosphors may not improve the WL emission. Hence, the low sensitivity of the human eye to red light emitted under low light conditions and the low brightness of red phosphors make critical-to-find red-phosphors suitable for white LEDs. Pumping of a mixture of blue, green-yellow, and orange-red emitting phosphors by UV-emitting LEDs is an alternative strategy to generate white light [42,43]. Down-conversion of blue LED photons to yellow and red photons wastes less energy than down-conversion of UV LED photons to blue, green, and red photons.

In order to simplify the design of a phosphor-converted WL emitting LED, a single-component white emitting phosphor rather than a multicomponent one is a very attractive strategy [44]. In this respect, doping and dopant-type, as well as dopants acting as spectral converters, play a key role.

In regard to the emission processes, down-conversion is the most commonly occurring process in devices that use near-UV or blue LEDs pumping down conversion materials [45]. However, proper crystalline hosts doped with RE trivalent lanthanide elements may yield efficient up-conversion luminescence and tunable multi-color emission [21,46]. In this context, RE ions are very attractive dopants due to the localization and shielding of their 4f states by the outer 5s and 5p subshells, the richness of energy levels, the characteristic emission energy and sharp emission lines, the long-lasting luminescence lifetime, the high color purity and possibility to combine different REs (co-doping) at different ratios to carry out tunable luminescence, and up-conversion processes [20].

To summarize, important critical technical issues in the field of inorganic phosphor-based WL LED-sources are the following [3,38,47,48,49,50,51]:

- available blue emitters are not efficient and stable enough,
- efficient emission in the green and yellow spectral ranges needs optimization,
- energy down-conversion by phosphors can involve reduced overall emission efficiency (Stokes losses) by photon reabsorption and emission in multilayer architectures,
- color shifts due to driving current and chip temperature,
- complex processing technology and careful design to balance the LED and phosphor(s) emission,
- the quality of the WL emission may depend critically on the phosphor amount and mixing of the combined LED emissions,
- the long-term stability of emitters,
- several processes competing with non-radiative loss channels and energy states affect the excitation and emission spectra of RE-based up-converter phosphors with decreasing quantum efficiency for increasing order of the multiphoton process,
- luminescence spectra that consist of a broad very-bright white emission band (380–780 nm) or yellowish light can be observed, that do not cover the whole visible-light range,
- strong reabsorption of the blue light by the red and green phosphors in the case of UV-LEDs pumping tri-color phosphors,
- generation of up-conversion WL by tri-doping of RE ions (usually Tm^3+^, Er^3+^, or Yb^3+^, for absorption of Yb^3+^ and energy transfer from Yb^3+^ to Er^3+^/Tm^3+^ ions) in oxide and fluoride hosts to overlap sharp red/green/blue emission lines has the major drawback that the up-conversion emission can be quenched by unexpected cross relaxation processes,
- the influence of processing, packaging, and assembly of the phosphor(s) on the device performance.

Among the suggested optimization approaches of WL-emitting LEDs there are laser diodes [52], mixing of inorganic phosphors and quantum dots [52,53,54], development of silicate-based phosphors [55], down-conversion mechanisms in combined blue-emitting nanowire LEDs with phosphors [56], and a phosphor-free approach based on nitride or ZnO white nanowire LEDs [57]. The market request of flexible non-flat WL sources has driven the research interest towards white phosphor-converted organic LEDs (OLEDs) and white OLEDs based on mixing different colored emitters [58,59]. Despite the advantages of OLEDs (such as low cost, compatibility with flexible substrates, and easy processing), they suffer from poor time stability, short-lifetime, and low-efficiency performances at the blue component [60]. Another investigated class of photo- and electro-luminescent materials is carbonaceous materials, including carbon nanotubes [61] and graphene [62,63,64,65].

## 3. Spectroscopy of Nanophosphors

Spectroscopy of nanophosphors is currently of worldwide interest, in the fundamental and applicative fields, favored by both the progress in the nanofabrication field and experimental observation pointing out changes of the general properties of lanthanide-activated phosphors introduced by nanoscale resizing. Chemical and physical techniques, such as, sol−gel, combustion, micro-emulsion, co-precipitation, laser ablation, ultrasonic spray pyrolysis, and template-based routes, are currently used for the synthesis of nanophosphors with control on size and shape, chemical composition, phase, doping amount, surface passivation, and functionalization steps [12,31,66,67].

Early experimental evidences of size-dependent spectroscopy of nanophosphors were remarkably increased photoluminescence quantum efficiency with high radiative efficiency (~20%) and luminescence lifetime enhanced of more than 5 orders of magnitude (ns versus ms) with respect to the bulk counterpart crystals in the case of ZnS nanocrystals doped with Mn (dopant content ranging from 1% for crystal size of 70 Ǻ to nearly 18% for crystal size of 3.5 Ǻ) [68]. Nanoscale-related emission enhancement and tunability were also reported for 20 nm size yttrium oxide (Y_2_O_3_) nanoparticles co-doped with Yb and Er ions [69] and for decreasing particle size (from 55 to 13 nm) due to the small Bohr radius of the exciton [25]. Enhanced emission efficiency can result from the combined effect between the energy levels of lanthanides and dopant-induced symmetry breaking of the host lattice favored by surface lowered coordination too [28,70]. Moreover, improved efficiency of up-conversion transitions was obtained in hexagonal and cubic NaREF_4_ (RE = Y, Pr to Lu) nanocrystals and NaYF_4_ nanoparticles co-doped with Yb, Er, and Tm by changing the shapes (polyhedra-, rod-, plate-, and dot-like) [71].

As previously outlined, the characteristic emission lines of RE elements are strictly related to their peculiar electronic configuration with the valence electrons 4f shielded from interactions with the host crystal lattice and ligands by means of outer shells. Unlike RE elements, the unfilled outermost more extended and delocalized d-orbitals of TMs, hybridize with ligand orbitals leading to splitting of the atomic (unperturbed) levels in the host crystal field. Hence, while the energy levels of the TM dopants are automatically influenced by the host crystals at any size-scale, spatial confinement has poor effects on RE-characteristic emissions stemming from intra-configurational f-f transitions. On the other hand, as the emission features of REs can be due to d-f transitions too, host-dependent energy-shifts of the RE emissions can be observed. Since the excited states of RE elements can have L–S coupling and J mixing, a single ^2S+1^L_J_ multiplet can describe the excited states of REs sensitive to the host crystal and yield host-dependent emission of RE centers [16]. Therefore, in nanophosphors, dopant-ligand distance and site symmetry may influence the emission from the excited states of RE dopants due to modified J–L coupling and J mixing effects with respect to the lower energy levels [16]. Moreover, changes in the emission efficiency can be induced by dopant-related symmetry breaking in the host material, that can be more effective because of site distortion favored by both the choice of dopant and surface lowered coordination [16].

In the framework of nanophosphor physics, tunable color output and multicolor emission as related to dopant content and dopant–dopant interaction, phase of the host dopant, dopant site symmetry, surface-to-volume ratio, phonon modes, and excited state dynamics are key topics. In this respect, generalities and examples will be provided in the following sections.

### 3.1. Tuning of the Color Output

**Figure 7:**
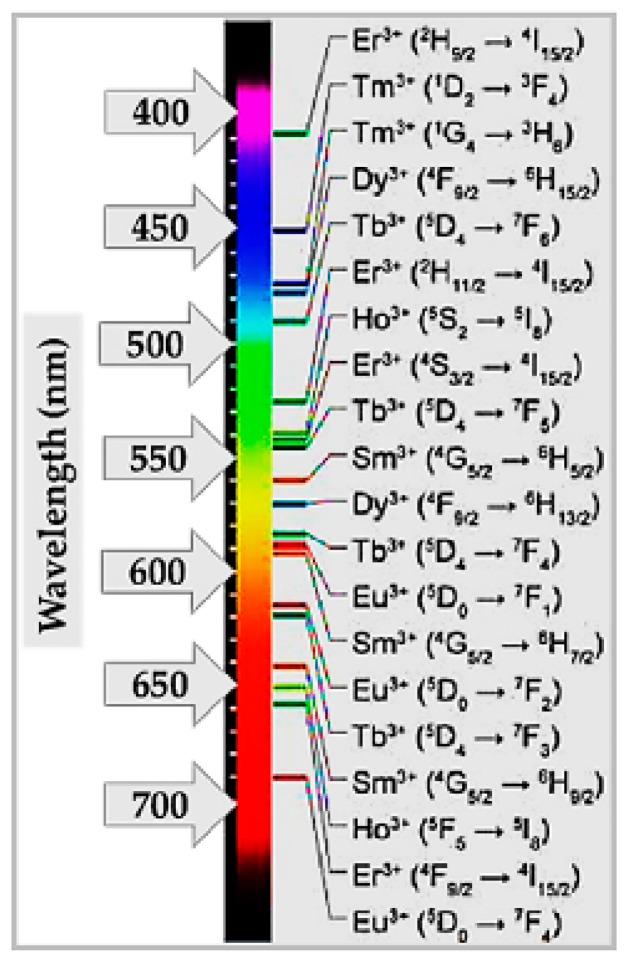
Diagram of the color output resulting from the main f-f transitions of RE elements.

For lighting applications of nanophosphors aiming at a simplified device design, broad range tunability of the color output and multicolor emission by single wavelength excitation are the main applicative purposes. In order to be able to implement this simple scheme, RE elements are key components due to their rich energy level diagram. As Figure 7 shows, multicolor output can be obtained by exciting different f-f transitions of the same RE element as well as by combining the emission from different RE elements.

The achievement of multi-peak tunable emission from nanophosphors requires proper choice and balance of dopant species and dopant−dopant interaction as well as a suitable host material and site symmetry due to the influence of lattice vibrations and crystal field on the radiative transitions of the dopants.

A single dopant emitter may allow the production of WL emission based on tuning of the intensity ratio between two different emission colors [72,73]. Alternatively, different dopant(s)–host combinations can yield multiple color emissions by means of balanced spectral overlap and relative ratio of different emission colors. Also, luminescence may be phase-dependent through the site symmetry of dopants in different host lattices and dopant–dopant interaction in multiple lattice sites [70].

**Figure 8:**
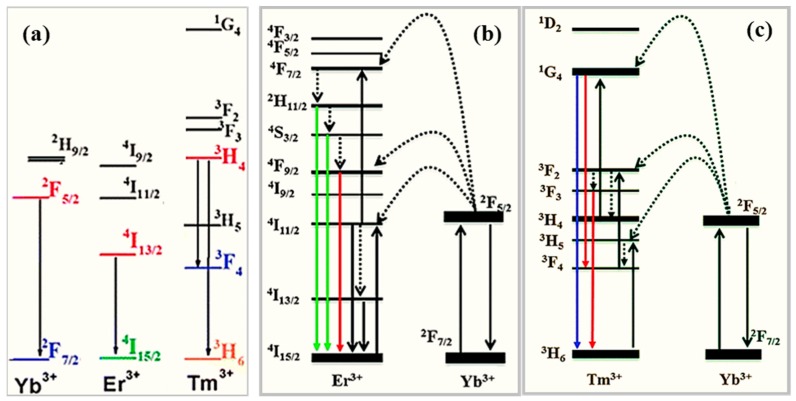
(**a**) Diagram of the energy levels of Yb, Er, and Tm and main radiative transitions. Scheme of the radiative emissions and energy transfer mechanisms of (**b**) Er-Yb pairs leading to green emission and (**c**) Tm-Yb pairs leading to blue emission.

An example of the effects on the emission spectrum due to the interplay between dopants is given by hexagonal phase β-YF_3_ nanocrystals co-doped with suitable contents of Er^3+^, Tm^3+^, and Yb^3+^ under excitation at 976 nm. Figure 8a shows the energy levels of Yb, Er, and Tm and their commonly exploited f-f transitions. Figure 8b,c depict the transitions and energy transfer processes occurring when Er-Yb pairs and Tm-Yb pairs are considered. The resulting emission is a combination of blue light emitted by the Yb^3+^/Tm^3+^ pairs, green light yielded from the Yb^3+^/Er^3+^ pairs, and red emission from the Tm^3+^/Er^3+^ pairs [74]. As a further example, the Dy^3+^ ion exhibits two main emission bands given by a characteristic blue emission (470–500 nm) and a yellow emission (560–600 nm) sensitive to the host material, with yellow-to-blue intensity ratio dependent on the Dy^3+^-lattice ligand covalent-bond length for Dy^3+^ replacing not equivalent lattice ions in oxides [75]. In the case of NaYF_4_ nanocrystals, with size ranging from 6 to 45 nm and co-doped with Yb^3+^ and Er^3+^ ions, green and red emission stem from Er^3+^ ions (Figure 8b) under near-IR laser excitation at 980 nm and it was reported that it increased red-to-green luminescence intensity ratio and reduced luminescence for decreasing size [76]. The red-to green ratio enhancement for smaller nanocrystals was ascribed to the influence of intrinsic phonon modes, vibration energy of surface ligands, solvent-mediated quenching, and surface defects [77,78,79]. Under increasing amounts of Yb^3+^ (25–60 mol %), the reduced distance between dopants was reported to favor back energy transfer from Er^3+^ to Yb^3+^ and, as a consequence of the reduced population of the excited levels of Er^3+^, a decrease in the blue and green emissions as well as emission color changing from yellow to red were observed [80]. A further example reporting the effects of the interplay between dopants is given by NaYF_4_ nanoparticles co-doped with Yb^3+^, Ho^3+^, and Er^3+^ that emit green light tunable from green to red and with intensity that can be enhanced by addition of Mn^2+^ [81,82].

**Figure 9:**
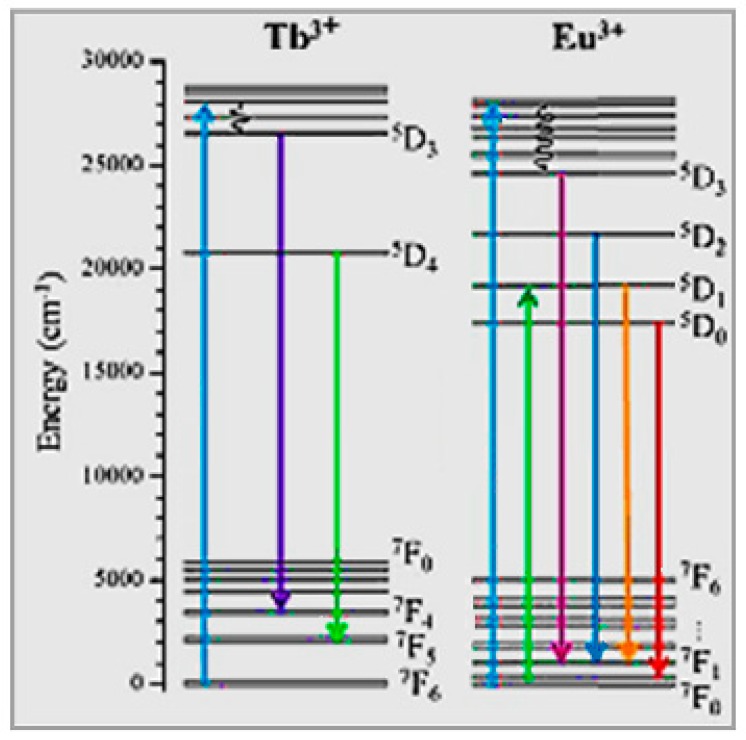
Energy levels of Tb and Eu. In the case of Tb-Eu co-doping, Tb can act as a sensitizer driving multicolor emissions from Eu.

Intense green and red emission peaks occur in LiYF_4_ colloids triply-doped with Eu^3+^, Tb^3+^, and Ce^3+^ at amounts of 13%, 14% and 1–5% respectively, due to Ce^3+^ and Tb^3+^ acting as sensitizer ions and the energy-transfer processes Ce^3+^ → Tb^3+^ and Ce^3+^ → Tb^3+^ → Eu^3+^ [83]. Figure 9, that shows the energy levels of Tb and Eu, sheds light on the role as sensitizer of Tb and multicolor emission from Eu^3+^ that can yield red color output with orange-red/red emission (due to the characteristic ^5^D_0_ → ^7^F_1_ (555 nm) and ^5^D_0_ → ^7^F_2_ (614 nm) transitions). Moreover, output color can be tuned from green to orange for increasing Eu^3+^ content. Interestingly, in the case of LiYF_4_ nanoparticles doped with Eu^3+^ ions, a phase transformation from tetragonal to orthorhombic is driven by increasing the content of Eu^3+^ activator ions, with the best luminescence performances carried out for Eu concentration of 15 mol %. Because of the occurrence of a concentration threshold of Eu, energy transfer processes enhancing Eu^3+^ emission in LiYF_4_ nanocrystals can be induced by addition of a sensitizer, such as Ce [84].

### 3.2. The Role of the Dopant Content

The dependence of the output light on the dopant amount is a way to tune multicolor emission and the relative emission intensity. For instance, in the case of NaYF_4_ nanocrystals doped with Yb, Tm, and Er (Figure 8), for a concentration ratio Yb^3+^/Tm^3+^ set to 20/0.2 mol %, the color output can be changed from blue to white by means of Er^3+^ content increased from 0.2 to 1.5 mol % [80]. Under excitation with a 980 nm wavelength, LiYF_4_ nanocrystals co-doped with Yb^3+^, Tm^3+^ (0.5%), and Er ^3+^ (0.1%, 0.2%, 0.3%, 0.5%) exhibit blue and green emission with green-to-blue ratio of the photoluminescence intensity dependent on the Er^3+^ concentrations [85]. Yttrium oxide nanoparticles co-doped with Er^3+^, Tm^3+^, and Ho^3+^ ions, show red-to-green emission ratio changing versus particle size and up-conversion output color tunable from blue to red upon changes of the dopant amount [86].

In cubic phase α-NaYF_4_ nanocrystals doped with high concentrations of Tm^3+^ and Yb^3+^ ions, energy transfer processes from Er^3+^ to Yb^3+^ (Figure 8b) impact on the relative intensities of the blue, green, and red emissions from Er^3+^, leading to color output tunable from yellow to red for Yb^3+^ amounts increased from 25 to 60 mol % [80].

In nanophosphors, dopant concentration plays an important role not only in multicolor tuning but also in luminescence quenching. In bulk phosphors, concentrations of the localized centers above a critical host-dependent value implies luminescence quenching typically due to cross-relaxation and energy-transfer mechanisms [20,72]. This phenomenon is termed “concentration-quenching” and occurs at dopant amounts of nearly molar 1%. From this lower limit up to molar 10–20%, luminescence continuously decreases up to orders of magnitude until disappearing. Luminescence quenching also occurs in nanophosphors with the peculiarity that the concentration threshold of dopants for observing suppressed luminescence is higher than in the bulk counterpart phosphor [87]. Notably, luminescence increasing rather than decreasing with increasing dopant content was reported in nanoparticles: Y_2_O_3_ doped with a Tb^3+^ shows concentration quenching at concentrations above ~5% in large sized particles and luminescence intensity increasing even at the dopant amount of 50% in as small as 3 nm (“as synthesized”) sized nanoparticles [88]. In order to explain this experimental evidence, reduced probability of ion–ion energy transfer due to out of resonance conditions and the absence of phonon modes able to participate in the energy transfer process were addressed [89].

To overcome concentration quenching in nanocrystals, lattice confinement of dopants was proposed, that is distribution of Yb^3+^ ions with very high content (98 mol %) arranged in well-defined order and structure in an orthorhombic lattice were found to generate a four-photon-promoted violet up-conversion very intense emission from Er^3+^ ions [90].

### 3.3. The Role of Surfaces

Other effects accounting for the differences in the emission issues between bulk and nanoscale phosphors are surface-to-volume ratio, surface-related defects, and disordering [91]. In general, the surface-to-volume ratio influences the fraction of the weakly coordinated surface dopant ions versus the strongly coordinated inner dopant ions. Also the contribution of defects, contaminants, and solvent/surfactants in wet chemical synthesis approaches has to be taken into account for reducing the size. Energy transfer being dependent on the ion–ion spacing and the critical distance for observing energy transfer being of the order of several nanometers, confinement and dopant amounts are expected to impact on the control of energy transfer in nanophosphors.

For instance, in NaYF_4_ nanocrystals co-doped with Yb^3+^ (20 mol %) and Er^3+^ (2 mol %), 980 nm excitation energy is effectively absorbed by the Yb^3^+ ions and transferred to the nearby Er^3+^ ions that exhibit three emission bands (525, 542, and 655 nm) due to multiphonon relaxation processes bridging different excited states (Figure 8b). Both total emission intensity and the green-to-red intensity ratio increase for nanocrystal size increasing above 50 nm because of a decreased quenching of the up-conversion emission from surface defects and ligands [92]. In turn, red and green color emissions from weakly coordinated surface dopants play a dominant role for nanocrystal size scaled down to a few nanometers (ultra-small nanophosphors). On this basis and for biomolecule labeling purposes, synthesis approaches of nanophosphors need to control structural order, phase, phase purity, dopant content, structure (core-shell), and size for decreasing size down to tens of nanometers [93,94,95,96].

In order to circumvent luminescence quenching due to surface quenching for decreasing size, confinement of dopants by a core-inert shell architecture is a valid strategy that limits the contribution of surface quenching species, enables better stability in water media and reduced fluorescence quenching due to adsorbed hydroxyl species, suppresses cross-relaxation and energy-transfer dopant–dopant interactions, as well as enhances the up-conversion efficiency [94,95,96,97,98,99].

For example, sub-10 nm-sized LiYF_4_ nanocrystals co-doped with Yb^3+^-Tm^3+^ pairs or Yb^3+^-Er^3+^ pairs showed enhanced photoluminescence and color emission (bright blue, sky blue, aqua, aquamarine, and green) tunable by dopant amount when encapsulated by an outer LiGdF_4_ shell [94,95,96,97,100]. Luminescence enhanced up to 30 times was found in the case of 8 nm NaYF_4_ nanocrystals, co-doped with Yb^3+^ and Tm^3+^ ions, after their embedding by a 1.5 nm thick NaYF_4_ shell [94]. For a NaGdF4 matrix having the core doped with Yb^3+^-Tm^3+^ pairs (Figure 8c) and the shell doped with Eu^3+^, excitation by 980 nm wavelength induces multi-step energy transfer from core to shell (through the transitions Yb^3+^ (core) → Tm^3+^ (core) → Gd^3+^ (core and shell) → Eu^3+^ (shell)) mediated by Gd^3+^ and strong red emission from Eu^3+^ results. Notably, in the absence of the core-shell architecture, the coupling among the different ions quenches the emission and up-conversion of Eu^3+^ ions [99]. Modulation of the up-conversion emission was also demonstrated in core-shell designs under pulsed laser pumping that controls the energy transfer between dopants through pulse duration [101].

When the core and shell of a nanophosphor are doped with different ions, the spatially confined energy transfer process can be exploited to manipulate the emission by modulation the ion–ion interaction. For instance, a LaF_3_ core-shell system with Eu^3+^-doped core and Tb^3+^-doped shell separated by an un-doped LaF_3_ shell enables control of the energy transfer between Eu^3+^ and Tb^3+^ (Figure 9) and the Tb^3+^/Eu^3+^ emission ratio by variation of the thickness of the LaF_3_ shell [101,102,103].

**Figure 10:**
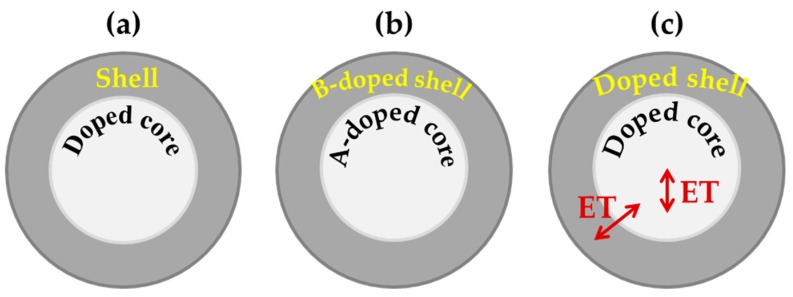
Schematics of the most common core-shell architectures: (**a**) passive-shell design, (**b**) active-shell coating design where A and B refer to different dopants, and (**c**) energy transfer (ET) core-shell design.

To summarize, in general, core-shell designs can be classified according to three main categories (Figure 10): (i) passive-shell design, where dopants are incorporated in the core [97], (ii) active-shell coating design, where dopants of the core and shell regions are different [98], (iii) energy migration core-shell design, where an optically active sublattice initiates energy transfer processes through the core-shell interface [99]. Other strategies to control surface-related effects involve assembly of nanophosphors with different chemistry and coupling between plasmon nanoparticles and lanthanide-activated nanophosphors [104].

### 3.4. Phonon Modes and Excited State Dynamics

At the nanoscale regime, the density of phonon states changes from a continuous (Debye approximation) to a discrete distribution, exhibits a size-dependent cutoff frequency for phonon modes and is unchanged for high frequencies phonon modes [89,105,106,107]. Since the low-frequency phonons contribute effectively to non-radiative relaxation processes between closely spaced energy levels, a lack of low-frequency phonon reduces losses and affects the luminescence dynamics of optically active ions. Whenever the low-energy phonon modes are absent, complete direct phonon relaxation between the levels with an energy gap less than the cut-off phonon frequency is completely suppressed. Hence, whereas spatial confinement has poor impact on the non-radiative decays yielding the emission of high-frequency phonons, less non-radiative relaxation channels result from a lowered number of allowed phonon modes: This means that only non-radiative relaxation involving electronic states with energy gaps close to or higher than the Debye energy of the lattice are size-independent. Therefore, in general, the electron–phonon interaction is size-dependent. Thermal relaxation is size-dependent too. Indeed, as a consequence of the low energy phonon modes being discrete, following excitation thermal equilibrium of nanoparticles may be inhibited because of no low-energy phonon state available to non-radiatively depopulate an excited level of an emitting center [108,109]. Hence, a level that in bulk material would fast relax, at the nanometer-scale retains its population long enough to decay radiatively rather than by the one-phonon decay.

The size-dependent electron–phonon interaction also influences energy transfer dynamics because energy transfer occurs by migration of electronic excitation between sensitizer-activator by resonant processes and/or by phonon-assisted transfer [110,111]. On the basis of the energy transfer mechanism, as the doping content increases, the reduced average ion–ion spacing implies increased probability per unit time of energy transfer. Turning to the nanoscale-regime, since the probability that the donor can find nearby acceptors decreases, the numbers of acceptors and donors get smaller and defect sites act as traps, negatively impacting on the efficiency of energy migration across dopant sites. Indeed, in a nanoparticle, core and surface atoms exhibit different coordination and symmetry of the surroundings, meaning that distorted surface bonds introduce defects and energy levels shifting as compared to the ones of the same core counterpart. This condition of out of resonance between energy levels of core and surface ions implies it requires emission or absorption of at least one phonon to bridge the energy difference [89,107].

Another important consequence of the size confinement of lanthanide dopants is the change of the excited state dynamics (emission decay and rise time, line broadening and line shift). Electron–phonon coupling plays a role in affecting the width of emission lines in nanophosphors. In general, homogeneous line broadening results from temperature-dependent phonon coupling (weak ion–phonon coupling) and electron–lattice interactions implies temperature-dependent line shifts. Based on theoretical considerations, size-dependent broadening and shifting of the spectral lines can be likely observed only in the limit of low temperatures (depending on the phonon density of states and occupancies of the phonon modes) and particle size much smaller than 50 nm (due to inverse relationship between the electron–phonon coupling and size) [89]. In regard to the emission decay-patterns of lanthanide-activated nanophosphors, which characterize the lifetime and decay mechanism of the optical center, deviations from the conventional exponential law of bulk phosphors and multi-exponential decay patters were observed. These behaviors were ascribed to several effects, such as intrinsic phonon modes, vibration energy of surface ligands, solvent-mediated quenching, differences in the probability of non-radiative decay of near-surface outer- versus inner-core ions, surface water, surface interactions, surface defects and inhomogeneous distribution of dopants.

Further effects of the spatial confinement and its influence on electron–phonon coupling are: (i) modified temperature-dependence (from T^7^ to T^3^) of the linewidth of characteristic transitions of Eu^3+^ in Y_2_O_3_ and Eu_2_O_3_ nanoparticles [105,112], (ii) increased lifetime (from 220 ns to 27 μs) of a multiplet of Eu^3+^ in Y_2_O_3_ nanocrystals, as compared to the bulk counterpart [113], and (iii) size-dependent elimination of direct phonon relaxation in Er^3+^-doped Y_2_O_2_S nanocrystals (anomalous thermalization) [105]. In addition, the vibronic coupling strength of vibronic transitions (i.e., radiative emissions concurrent with the absorption or emission of at least one phonon) is expected to change turning from bulk phosphors to nanophosphors [89].

Upon decreasing nanoparticle size, an enhanced or decreased decay time can result depending on doping [114,115]. While for large ion–ion spacing (diluted doping) decay profiles satisfy a single-exponential law with a lifetime comparable to the one of the un-doped counterpart, photoluminescence intensity decays non-exponentially for increasing amount of the luminescent ions [116]. Non-exponential decay time and shortened decay time can be favored by increasing excitation intensity [117]. In nanophosphors, the radiative lifetime of dopant states can be affected by change of the refractive index due to size tuning and modified surroundings [118] and field oscillations that implies spontaneous emission inhibited for particle size ranging from 100 nm to 2 μm and, conversely, enhanced lifetime of spontaneous emission at the sub-wavelength regime of nanophosphor size [44].

## 4. Nanoscale-Related Unconventional Production of White Light

Among designs of WL-emitting LEDs, the most common ones exploit a single-phase host material: (1) singly doped with a lanthanide ion having a complex energy level structure able to emit multiple colors (for instance, Sm^3+^, Eu^3+^, or Dy^3+^), (2) co-doped with red, green, and blue emitting or yellow and blue emitting RE ions, (3) including ion–ion pairs (for instance, Ce^3+^/Mn^2+^, Ce^3+^/Tb^3+^, Ce^3+^/Ho^3+^, Tb^3+^/Sm^3+^, etc.) participating in energy transfer processes, (4) triply-doped with ions emitting different colors (Ce^3+^/Li^+^/Mn^2+^, Eu^3+^/Tb^3+^/Tm^3+^, Eu^2+^/Tb^3+^/Eu^3+^) [44]. A survey of the literature enabled the observation that WL emission results from materials at least doubly-doped with different RE ions or including RE ions as stoichiometric components. For instance:

- nano-sized compounds incorporating Nd as a dopant or a full stoichiometric component emit anti-Stokes wide band emission [44,117,119,120,121,122],
- Yb-doped: Y_3_A_l5_O_12_ (YAG) nano-crystalline ceramics emit bright anti-Stokes WL [123],
- anti-Stokes WL emission is yielded by YAG (Yb_3_Al_5_O_12_) and (Yb,Y)_2_O_3_ nano-powders [124], LiYbF_4_ nanocrystals [125], (Yb^3+^,Ln^3+^, Tm^3+^, or Ho^3+^)-doped YVO_4_ powders [126], un-doped and Er-doped LiYbP_4_O_12_ nanocrystals [127,128],
- under near-infrared excitation excitation in vacuum, single compound lanthanide oxides (Yb_2_O_3_, Sm_2_O_3_, CeO_2_) [129,130] and Yb_3_Al_5_O_12_, (Yb,Y)_2_O_3_ crystals [124] emit WL by up-conversion mechanisms,
- Er_2_O_3_ emits up-conversion luminescence by band-to-band multiphoton excitation under vacuum and near-IR excitation [131],
- bright up-conversion WL is emitted by Ln-doped LaF_3_ nanoparticles [132] and LaF_3_ nanoparticles co-doped with Tm^3+^, Tb^3+^, and Eu ^3+^ [133],
- white LEDs were designed based on oxynitride or nitride phosphors doped with RE elements [134,135],
- luminescence with high quantum efficiency values, comparable to conventional crystalline phosphors, was observed under excitation with deep-UV light of RE-free transparent phosphate glasses [136],
- a red phosphor to be used as a high-power warm WL-emitting LED was obtained by a RE-free approach based on Mn^4+^ and Mg^2+^ doped BaMgAl_10_O_17_ [137].

Alternatively, under monochromatic IR-excitation, first WL broadband emission was observed from a nominally un-doped oxide nanomaterial (i.e., in the absence of luminescent RE centers as main components) and in the presence of a TM-dopant independently on the temperature.

To detail, WL emission was demonstrated in the following nanoscale-sized materials:

- Cr-doped Gd_3_Ga_5_O_12_ (GGG) nano-powders excited by a continuous wave (CW) laser diode emitting at 803.5 nm [18],
- nominally un-doped yttrium oxide (Y_2_O_3_) and Nd-doped (up to 20%) Y_2_O_3_ nano-powders excited by a CW laser diode emitting at 803.5 and 975nm [17,19,138],
- yttrium silicate (γ-Y_2_Si_2_O_7_) and Yb^3+^/Er^3+^/Tm^3+^ triply-doped Y_2_Si_2_O_7_ nano-powders excited by a CW laser diode emitting at 975 nm [139,140,141,142,143],
- erbium oxide (Er_2_O_3_) nano-powders excited by a laser diode emitting at 800 and 975 nm [144].

Experimental details about materials and synthesis methods of the abovementioned samples are detailed in the cited literature and are not reported here for the sake of brevity and because the focus of the discussion is the observed WL emission characteristics.

A first remark is that the observed WL emission was found to be a nanoscale phenomenon in that spectroscopic investigation of the bulk counterpart materials did not point out the generation of WL. This important finding means that any upcoming luminescence model formulated to describe this occurrence must take into account the nano-regime as basic background. Other key points are the unknown origin of the reported WL emission and the identification of its both common and unconventional behaviors as compared to the existing literature.

In regard to the first point, a critical discussion of the experimental findings was reported in the case of Y_2_O_3_ nanoparticles [19]. Turning to the comparison with the existing scenario, it is a common characteristics of the existing WL emission strategies and unconventional WL emission that WL intensity and generation can be tuned by pressure, excitation intensity and wavelength, chemical environment (gas background, doping, surface effects), size, and temperature, according to the following experimental evidence. In general, the emission intensity depends on both the background pressure [129] and pumping power: the threshold pumping power to produce WL emission decreases for decreasing background pressure from atmospheric to vacuum pressure and, for given laser power, a reduction in the pressure favors WL emission [122,123,124,126,127,129,145].

For instance, studies dealing with Er_2_O_3_ nanoparticles with cubic phase and size ranging from 15 to 80 nm pointed out, under excitation at 808 and 975 nm and background pressure ranging from 0.03 mbar to 1 atm, a broadband (400–900 nm) WL emission band with a strong dependence on the laser pumping power leading a threshold pumping power of 4 W at low pressure and spectral shift of the emission peak at atmospheric pressure [144]. 

**Figure 11:**
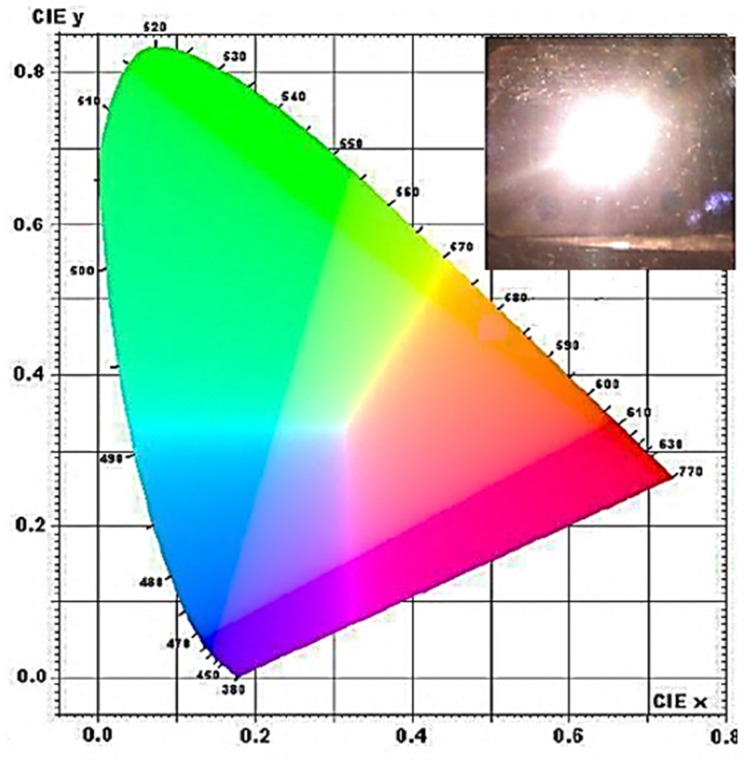
CIE color diagram and point of the color space (black circle) corresponding to the color coordinates of the WL emission (shown in the inset) observed by un-doped Y_2_O_3_ nano-powders.

In the case of un-doped Y_2_O_3_ nano-powders, the threshold pumping power was found to depend on both background pressure and excitation wavelength. That is, in the case of the 803.5 nm excitation wavelength, the WL emission was easily observed under pressure conditions of 0.02 mbar at a pumping power of 0.7 W and, under atmospheric pressure conditions, a pumping power value of 1.1 W was needed to induce generation of WL broadband emission. Turning from 803.5 nm to 975 nm excitation wavelength, a threshold pumping power of 6 W was measured under pressure conditions of 0.02 mbar and no emission at atmospheric pressure [17]. On the other hand, the threshold pumping power was observed to decrease for increasing Nd-dopant amount: for the 20% Nd content, WL emission was obtained at a pumping power as low as 0.12 W under 0.02 mbar and became detectable at 0.73 W under atmospheric pressure [17,138]. The WL emission characteristics of the Y_2_O_3_ nanoparticles investigated in terms of the lighting standard parameters indicated that the better performances result under laser excitation of 803.5 nm and in the absence of Nd-doping. Interestingly, WL emission with chromatic properties very close to the ones of commercial incandescent bulb sources was measured (Figure 11): a color rendering index (CRI) = 99 (to be compared with the theoretical limit 100), correlated color temperature CCT = 2756 K (to be compared with 2856 K of the color point in the International Commission on Illumination (CIE) color space that marks the chromaticity of artificial light sources most comfortable for the human eye) and efficiency higher than a 90 W incandescent lamp source [17].

Notably, the experimental findings indicated that, although the presence of Nd favors the generation of WL emission, nominally un-doped Y_2_O_3_ nanoparticles may efficiently emit WL in the absence of RE-emitting centers too. This is an unprecedented experimental finding that suggests a host-related mechanism leading to WL emission. By comparison, short-living and stable broadband WL emission were measured in the case of GGG and Cr-doped GGG, respectively. Hence, depending on the host crystal and nature of the dopant (RE versus transition metal element) dopant-related mechanisms are expected to play a role in promoting WL emission that needs proper investigation.

Once the wavelength, background pressure, and pumping power conditions to observe WL emission are determined, the emission intensity can be expressed as a power function of pumping intensity, that is I = AP^Nph^, where I is the emission intensity, A is a constant, and N_ph_ is the number of incident-absorbed photons [146]. A high order N_ph_ of the process and sigmoidal shape of the emission intensity versus the pumping power are characteristic for a photon avalanche process [120,121,123,124,127,147]. On the other hand, an exponent N_ph_ lower in vacuum than under atmospheric pressure was reported [121].

In agreement with the available reports, a threshold pressure for detecting measurable intensity and a non-linear increase of the WL emission intensity with increasing incident laser power was observed [17,139]. Above a certain power threshold, the increase in intensity followed a power law that is indicative of multi-photon absorption processes and this issue was discussed as related to electronic state filling up and saturation of energy states at higher energy with blue-shift of the emission energy for increasing pumping power [17,139,148]. Interestingly, in the case of un-doped Y_2_O_3_ nanoparticles the presence of more than one value of the order of the process depending on the interval of pumping power was observed [19]. Similar behavior was measured in Yb-doped Y_2_Si_2_O_7_ nano-powders and was absent in the un-doped Y_2_Si_2_O_7_ host nanocrystal [143]. A tentative explanation of the experimental results was provided for un-doped Y_2_O_3_ based on the band structure picture that predicts an offset between optical and electronic band gaps due to dipole-forbidden transitions occurring in the ideal bixbyte structure that become possible in conditions of reduced symmetry [19].

As discussed about the spectroscopic properties of nanophosphors, scaling down sizes to the nanometer level impacts on the luminescent properties of insulating inorganic phosphors. While the surface effects can broaden the emission/absorption lines and cause non-exponential decay patterns of the luminescence intensity [149], changes in the electronic dispersion can correspond to increasing luminescence decay with the reduction of the average grain size [69,118,150,151]. For doped phosphors, decreased size or grain size result in reduced local crystal symmetry with the optically active surface ions that occupy the sites of lower symmetry. All of this can lead to size-dependent optimal doping levels [87], enhanced temperature sensitivity [152], improved luminescence efficiency [153], decreased emission intensity [115], and changes in the decay patterns. In this respect, the reduction of the phosphor size and grain size may induce opposite effects (enhanced or decreased) decay time depending on the material (dopant and dopant content) [114,154].

In regard to concentration quenching, WL emission was observed in Nd-doped Y_2_O_3_ up to 20% Nd concentration [17]. By comparison, in the counterpart bulk material, above a threshold Nd content of a few percentage points, quenching of the emission intensity occurs due to energy transfer between Nd-adjacent centers [155,156]. Size-dependent studies of the doping concentration optimal for peak luminescence intensity demonstrated that, while reducing the phosphor size, at least doubled dopant content, as compared to the bulk counterpart, causes concentration quenching [87]. This evidence is consistent with efficient luminescence still occurring for high dopant content from Nd (20%)-doped Y_2_O_3_ and Yb (20%)-doped Y_2_Si_2_O_7_ nanocrystals [143].

Also, in inorganic nanophosphors, emission decay and rise patterns are sensitive to dopant content, pumping power, background pressure, and temperature. Whereas for very diluted doping conditions (i.e., far apart dopant ions) decay profiles are commonly single exponential with a lifetime comparable to the value of the un-doped single crystal, increased content of the luminescent ions can result in non-exponential decay behavior of the photoluminescence intensity [116]. At low temperatures, the non-exponential behavior of emission decay patterns can result from broken or lowered symmetry by the presence of surface dopant ions and heterogeneous distribution of the core dopant. In this case, a single-exponential decay profile can be recovered by increasing the particle size, meaning improving crystal order.

In regard to Cr-doped GGG, the emission intensity of the visible broadband WL emission increases slowly versus increasing excitation pumping power up to 2.2 W, where slowly means a few seconds to achieve a steady-state value. To induce a faster rise time, the pumping power needs to be further increased. In turn, the emission intensity decays in a non-exponential way with a decay time strongly dependent on the background pressure, that is changing from 50 to 150 ms for pressure varied from 21 to 0.032 mbar [18]. 

For un-doped Y_2_O_3_ nano-powders, the accumulated experimental evidence demonstrated that the decay and rise patterns are not sensitive and very sensitive respectively, to the pumping power, in that the delay for the intensity rising to its maximum is shortened for increasing pumping power. [17,148,149]. Upon the addition of Nd (20%) dopant, the emission intensity progressively increases under increasing the pumping power. By comparison with the literature, a relatively slow (of the order of seconds) build-up time of the up-conversion photoluminescence was reported at low excitation power with shortening of such build-up time for increasing pumping intensity [121,125,129,157,158].

Turning to the impact of the dopant content and nanophosphor size on the rise and decay patterns, for Nd-doped Y_2_O_3_ nano-powders, experiments point out the following evidence [17,148,149]:

- the decay patterns of the WL emission do not vary remarkably versus the Nd concentration changed from 2% to 20%, which is consistent with the observed host-related WL emission,
- the onset of the WL emission is strongly dependent on Nd content below 10% of dopant concentration where there is a delay before the rise of the WL to its maximum value lasting more and more for decreasing dopant concentration,
- for Nd (10%)-doped Y_2_O_3_ nano-powders, the rise pattern depends on the particle size in that its rising profile is steeper and the intensity increases faster for decreasing particles size,
- for Nd (10%)-doped Y_2_O_3_ nano-powders, size-independent patterns were observed in the 20 to 50 nm size-range as well as decay time slower with increasing size up to 250 nm.

For materials in which cross-relaxation between luminescent ions is limited due to energy mismatch, exponential luminescence decay can occur [154,159,160,161]. Increasing excitation intensity was reported to cause an increasing non exponential behavior of the decay time as well as shortening of the effective average decay times [117] which is in agreement with experimental findings about the unconventional WL emission from Nd-doped Y_2_O_3_ [17,148]. Therefore, the occurrence and spectral distribution of the WL emitted from Nd-doped Y_2_O_3_ was not strictly related to/dependent on the dopant concentration and presence (even if Nd makes it easier to induce WL under low pumping power and higher environment pressure conditions) as well as the observed non-exponential decay patterns almost insensitive to dopant concentration and particle size below 50 nm, as well as WL emission up to 20% of Nd concentration in Y_2_O_3_ [17], are consistent with WL emission stemming from the nominally un-doped oxide matrix.

Temperature is another important parameter affecting the spectroscopy of inorganic phosphors. Photoluminescence intensity can decrease for rising temperature because of the probability of non-radiative transition being higher for increasing temperature. On the other hand, it is reported a temperature-independent emission profile over a wide range (from room temperature to 77 K) at fixed pumping intensity [121] as well as emission intensity that increases as the temperature rises and due to vibronic contribution [162,163]. In our experiments, un-doped and Nd-doped Y_2_O_3_ nano-powder samples, excited by a laser diode emitting at 803.5 nm with pumping power of 2W, show broad-band (400–900 nm) WL emission spectra independent on the temperature over a range from 35 to 300 K. Instead, dynamic properties, such as the decay rate, were found to be temperature-dependent with faster decay for falling down temperature [17].

Another peculiar result of the observed unconventional WL emission is temperature-independent WL emission spectrum of Cr-doped GGG [18]. In this respect, to characterize the optical behavior carefully, low-temperature (i.e., around 10 K) spectroscopic measurements were performed to avoid thermal broadening of the spectral bands and disclose optical features related to the crystal-field allowed transitions of Cr as well as the temperature-dependence of the vibronic emissions. Spectra of Cr-doped GGG reported in the literature under different excitation conditions [145,164,165,166] indicate that Cr-doped GGG emits a featured spectrum with narrow lines stemming from the luminescence of Cr^3+^ ions and vibronic side bands up to nearly 200–300 K. For increasing temperature, any structure is lost and the spectra become broadband-like. Instead our investigation of Cr-doped GGG revealed a completely different behavior under excitation conditions not reported by the available reports (laser excitation at 803.5 nm and larger intensity of pumping power (2 W)): a broadband unstructured WL emission profile was measured over a wide temperature range (from 33 to 300 K) without structural transitions [18]. This experimental evidence deserves further investigation for fundamental understanding of the involved mechanisms and emission.

## 5. Conclusions

Solid-state lighting devices exploiting LED pumping sources to irradiate phosphors materials dominate the actual market of white light emitting artificial sources for indoor and outdoor applications. The market request of mercury-freedom, efficient power output, compact devices for integrated electronics, and high-performance white light emission has prompted both theoretical and applicative interest in nano-sized phosphors, termed nanophosphors.

In this review paper, we have overviewed the spectroscopy of phosphors with a focus on the main radiative and non-radiative mechanisms and the luminescence yield related to rare earth dopants. Moreover, we have discussed phosphor-activated LEDs by paying attention to the advantages and disadvantages of the actual designs, comparison with organic LEDs and future perspectives in the lighting field. In this context, the role of nanophosphors is key and promising because of both progress of nanofabrication techniques (that allow us control on size, shape, composition, phase, structure, and surface chemistry at the nanoscale) and the changes of the spectroscopic properties induced by nanoscale resizing as compared to the bulk world. In this respect, we have overviewed and discussed spectroscopy of nanophosphors by comparison with bulk phosphors and remarked the following issue: (1) the impact of the spatial confinement on rare earth emission depending on the host material and angular momentum coupling, (2) multicolor tuning by varying the content of the emitting center and by the interplay between energy transfer and up-conversion processes depending on the proper combination of activator-sensitizer ions, (3) effects of the discrete density of state of phonons and its low energy phonon cutoff on non-radiative and radiative emission, and (4) how dynamical quantities (such as probability of radiative and non-radiative transitions, lifetime of the excited states, thermal line broadening and thermal line shifting) are affected by the nanoscale-regime.

As a further experimental evidence of the fascinating behavior of nanophosphors, we have reported on the observed emission of warm broadband white light emission from nominally un-doped oxides (Y_2_O_3_ nano-powders) and TM-doped garnet crystals (Cr-doped GGG). This phenomenon is unconventional in that the existing strategies to obtain WL sources mainly include RE-dopants and single-phase compounds containing REs as stoichiometric components. Peculiar interesting properties of the disclosed unconventional white light emission are: (1) its occurrence is strictly related to the nanoscale-regime, (2) although it may be favored by the presence of a dopant, its occurrence does not demand doping of the host oxide crystal, and (3) its inherent origin is still unknown and poses challenging questions in the field of luminescence at the nanoscale-regime.
